# In situ structure of actin remodeling during glucose-stimulated insulin secretion using cryo-electron tomography

**DOI:** 10.21203/rs.3.rs-2694866/v1

**Published:** 2023-04-04

**Authors:** Weimin Li, Angdi Li, Bing Yu, Xiaoxiao Zhang, Xiaoyan Liu, Kate White, Raymond Stevens, Wolfgang Baumeister, Andrej Sali, Marion Jasnin, Liping Sun

**Affiliations:** ShanghaiTech University; ShanghaiTech University; ShanghaiTech University; ShanghaiTech University; ShanghaiTech University; University of Southern California; ShanghaiTech University; Max Planck Institute of Biochemistry; University of California, San Francisco; Technical University of Munich; ShanghaiTech University

## Abstract

Actin mediates insulin secretion from the pancreatic β-cell through a remodeling process. Previous studies have been hampered by limited resolution, providing an ambiguous depiction of actin remodeling as a process that begins with depolymerization into actin monomers, followed by repolymerization into actin filaments. Here, we report the in situ structure of actin remodeling in INS-1E β-cells during glucose-stimulated insulin secretion at nanoscale resolution. We demonstrate that actin remodeling occurs at the cell periphery rather than in the cell interior. The actin filament network at the cell periphery exhibits three marked differences after remodeling compared to those under basal conditions. First, approximately 12%of actin filaments reorient, their angle changing from 0–45° to 45–90° relative to the plasma membrane. Second, the actin filament network remains predominantly as cell-stabilizing bundles but partially reconfigures into a less compact arrangement. Third, actin filaments anchored to the plasma membrane reorganize from a “netlike” to a “blooming” architecture, featuring radial projections emanating from their anchor points. Remodeling precedes the transport of insulin secretory granulesto the plasma membrane and their release from it. Furthermore, the density of actin filaments and microtubules around insulin secretory granules is lowered after remodeling compared to the basal conditions, as expected for the subsequent granule transport and release. Finally, actin filaments and microtubules are more densely packed than under basal conditions. These findings advance our structural and functional understanding of actin remodeling during glucose-stimulated insulin secretion in pancreatic β-cells.

## Introduction

Actin filaments are essential for a variety of cellular functions, including maintaining the cell structure, driving cell migration and division, and governing the movement of subcellular components, such as mitochondria, lysosomes, and insulin secretory granules (ISGs)^1,2^. Cellular signaling pathways, such as Rho GTPase pathway and PI3K/Akt pathway, cause the remodeling of actin filaments^3,4^. An early 2D visualization of actin remodeling, starting with a parallel alignment and ending in an “unidentifiable dense mass”, was achieved *in vitro* for chemically fixed β-cells using double-contrasting electron microscopy in 19725. Since then, actin remodeling has been studied at an even lower resolution by fluorescence imaging with artificial fluorescence labeling and by western blot analysis, revealing that actin filaments first depolymerize into actin monomers (G-actin) and then repolymerize into actin filaments (F-actin)^5–10^. To date, the *in situ* structure of the actin filaments before and after remodeling has yet to be established at the nanoscale.

Glucose-stimulated insulin secretion (GSIS) in pancreatic β-cells is a biphasic process defined by the presence of two distinct pools of ISGs: readily releasable pool (RRP) and reserve pool (RP)^7^. ISGs in RRP are rapidly released within the first 5–10 minutes, corresponding to the first phase, followed by a prolonged second phase lasting hours^11,12^. Actin filaments are recognized as a key mediator of GSIS through observation of the restricted ISG movements during actin stabilization by drugs *via* fluorescence microscopy^8,13^. Through remodeling, actin filaments regulate the transport of ISGs from the cell periphery to the plasma membrane (PM) and the release of their contents into the extracellular space^8–10,14^. Under basal conditions, actin filaments act as a barrier parallel to the PM to block ISGs from approaching the PM^8,10^. In the first phase, actin filaments depolymerize into actin monomers, allowing the rapid release of ISGs in RRP^8,9^. In the second phase, actin filaments repolymerize into a new actin network that facilitates ISGs to transport to and release from the PM^8,9,13^. Nevertheless, quantifying the differences between the actin filament network before and after remodeling is imperative to understand the mechanisms underlying its blocking or facilitating role during GSIS.

During GSIS, the transportation of ISGs first involves long-range movement driven by kinesins on microtubules (MTs), with the subsequent handoff to myosin V for the short-range movement on actin filaments to the PM^15,16^. Using fluorescence microscopy, MTs have been reported to negatively regulate insulin secretion at the cell periphery under basal conditions^17^; it was proposed that direct interaction between actin filaments and MTs promotes the transport and release of ISGs in the second phase^10^. Due to the limited resolution of fluorescence microscopy, the structural basis of interactions among ISGs, actin filaments, and MTs during GSIS remains to be determined.

In this work, we used a multimodal imaging approach to visualize and quantify actin remodeling at both the periphery and interior of the cell under basal conditions as well as during the first and second phases of insulin secretion in rat INS-1E β-cells. First, we quantified changes in actin meshwork organization during GSIS using total internal reflection fluorescence (TIRF) microscopy and structured illumination microscopy (SIM). Next, we employed cryo-electron tomography (cryo-ET) to reveal the structure of actin remodeling at the nanoscale and variations in the volume and orientation of actin filaments during GSIS^18–20^. In addition, we quantified the arrangement of peripheral actin filaments before and after remodeling by their distances, and analyzed the alignment of peripheral actin filaments anchored to the PM *via* their distances and angles. Lastly, we characterized the interactions among ISGs, actin filaments, and MTs at the cell periphery by their distances. Based on these quantitative analyses, we establish the model for actin remodeling at the cell periphery, providing a more detailed and accurate depiction of changes in the architecture, alignment, and interaction of actin filaments with ISGs and MTs during GSIS in pancreatic β-cells.

## Results

### Multimodal visualization of actin remodeling during glucose-stimulated insulin secretion

We imaged the clonal INS-1E cells, a rat β-cell widely used to mimic the biphasic insulin secretion in β-cells in response to glucose stimulation^21–23^. We began by multimodal imaging to visualize actin remodeling during GSIS. We first employed TIRF microscopy (x and y resolution of ~120 nm) to capture the biphasic insulin secretion response over 40 minutes of glucose stimulation (Extended Data Fig. 1 and Supplementary Video 1). The first phase occurred during the first 10 minutes after glucose stimulation, while the second phase occurred between 10–40 minutes, as we recorded the intensity of the NPY-mCherry marker for ISGs over time. Actin stress fibers were observed at the basal membrane, maintaining the cell structure. We then performed a western blot experiment to detect the G-/F-actin ratio in the cell. We observed a significant increase in the ratio of the monomeric to filamentous actin states during the first phase, followed by a significant decrease during the second phase, indicating the remodeling of actin filaments during each phase (Extended Data Fig. 2).

**Figure 1:**
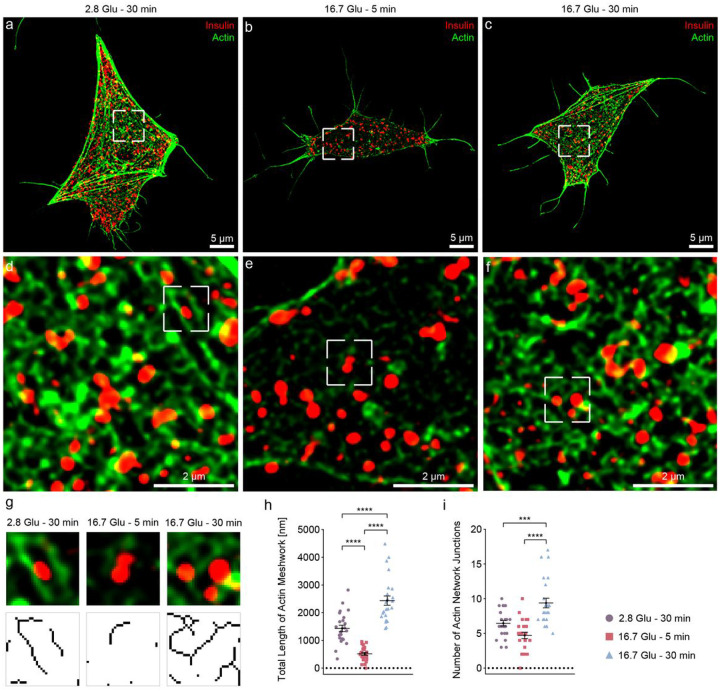
Visualization and quantification of the actin meshwork organization near the basal membrane during GSIS using SIM. SIM images of actin (green) and ISGs (red) under basal conditions (**a, d**) and 16.7 mM glucose stimulation for 5 min (first phase; **b, e**), and 30 min (second phase; **c, f**). **a, b, c** 2D projection of a 300 nm-thick volume from the basal membrane. **d, e**, **f** Zoomed-in views showing actin meshworks peripheral to ISGs. The images for subsequent skeletonization analysis are also highlighted with a small white rectangle of 0.97 um^2^. **g** Actin and ISG images on selected subsections and corresponding skeletonized actin meshworks. (n = 24, four sampled subsections on each cell, six cells used in each condition). For further analysis, only actin bundles with lengths longer than or equal to one pixel were considered. **h** Total length of actin meshwork in each subsection under different conditions. **i** Number of actin network junctions in each subsection under different conditions. * indicates p<0.05. ** indicates p<0.01. *** indicates p<0.001. **** indicates p<0.0001 by one-way ANOVA. A total of six INS-1E β-cells were prepared and used in three independent experiments for all conditions.

Next, we performed SIM (x and y resolution of ~30 nm) to assess the actin meshwork organization around ISGs in different subsections of the β-cell. We visualized the projections of actin and ISGs along the z-axis of a 300 nm-thick cell section near the basal membrane (Fig. 1a–c). We observed a number of bundles in the basal conditions (44% ± 6%, bundles area by total actin area) and the second phase (30% ± 3%), but few in the first phase of GSIS (18% ± 1%). Compared to the basal conditions, actin intensity significantly decreases in the first phase of insulin secretion, followed by an increase in the second phase (Fig. 1a–f). These changes are in agreement with changes in the G-/F-actin ratio measured by western blot (Extended Data Fig. 2). In addition, they are consistent with similar measurements by western blot and immunofluorescence techniques in previous studies^24,25^. To quantify the structural properties of actin meshwork organization during GSIS, we selected a few subsections of actin surrounding ISGs rather than other areas where actin forms bundles in the cell (Extended Data Fig. 3). After skeletonizing the actin meshwork based on the intensity of the actin fluorescence label^26–28^ (Methods, Fig. 1g), we quantified the length of actin meshwork and identified network junctions as connections in the actin meshwork. The actin meshwork is significantly shorter in the first phase (515 ± 53 nm) and significantly longer in the second phase (2435 ± 167 nm) compared to the basal conditions (1432 ± 116 nm), in line with the western blot measurements (Fig. 1h and Extended Data Fig. 2). Notably, actin forms a more complex network with an increased number of junctions in the second phase (9.4 ± 0.7) compared to the basal conditions (6.5 ± 0.4) and the first phase (4.7 ± 0.5) (Fig. 1i). In summary, these results characterize actin remodeling near the basal membrane during GSIS, including depolymerization in the first phase as well as repolymerization into a longer and more complex actin meshwork in the second phase.

Furthermore, we used cryo-ET to examine actin remodeling during GSIS at nanometer resolution, and quantify changes in architecture, alignment, and interaction of actin filaments with other subcellular components. We collected tomograms of the cell periphery (0–6 μm from the PM) and the cell interior (0–3 μm from the nuclear membrane) (Supplementary Table 2). The cell periphery of INS-1E β-cells is only~250 nm thick, and vitrified cells can be imaged directly using cryo-ET. To access the cell interior, vitrified INS-1E β-cells were first milled using a cryo-focused ion beam (cryo-FIB) machine to obtain lamellas with a thickness of approx. 150 ± 50 nm suitable for cryo-ET data collection^29,30^ (Extended Data Fig. 3). In total, we collected 42 tomograms for 34 cells, including 24 tomograms at the cell periphery and 18 tomograms at the cell interior under basal conditions (2.8 Glu - 30 min), in the first phase (16.7 Glu - 5 min), and the second phase of insulin secretion (16.7 Glu - 30 min) (Supplementary Table 1).

**Figure 2:**
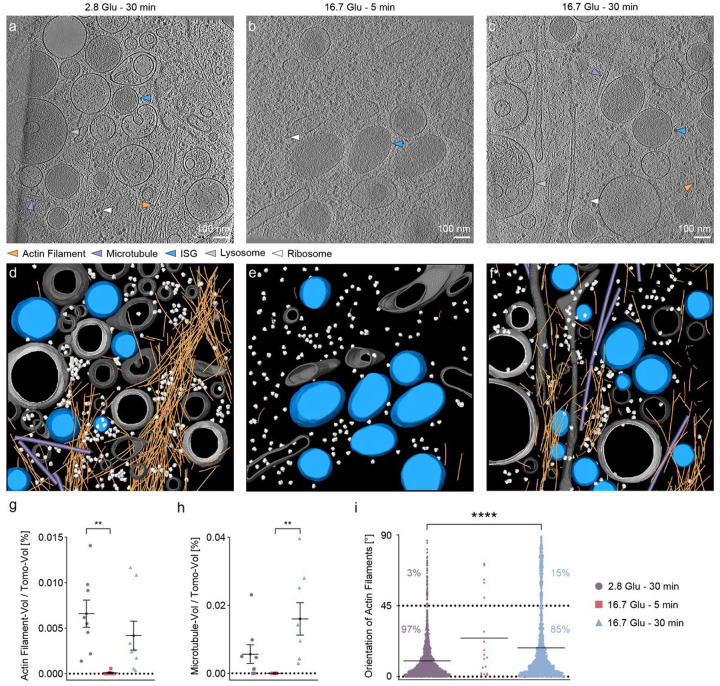
Quantitative analysis of actin filament and MT orientations at the cell periphery during GSIS. **a, b, c** Slices through a tomogram under basal conditions (2.8 Glu - 30 min; **a**), in the first phase (16.7 Glu - 5 min, **b**), and in the second phase (16.7 Glu - 30 min, **c**). Arrows indicate actin filaments (orange), MTs (violet), ISGs (blue), endoplasmic reticulum (ER; green), lysosomes (silver), and ribosomes (white). **d, e, f** Respective segmentations with the same color code. Non-ISG membranes are shown in gray. g, **h** Volume ratio of actin filaments (**g**) or MTs (**h**) to the tomogram in each tomogram under different stimulation conditions. **i** Orientation of actin filaments under different stimulation conditions. * indicates p<0.05. ** indicates p<0.01. *** indicates p<0.001. **** indicates p<0.0001 by one-way ANOVA. n = 24 tomograms, corresponding to eight tomograms for each condition from three biologically independent experiments.

In tomograms of the cell periphery, we distinguished various types of subcellular components, including actin filaments as single fibrous filaments^31,32^; MTs as rigid tubular structures^33,34^; mature and immature ISGs as membrane enclosed vesicles with and without dense core, respectively^35^; lysosomes as monolayer vesicles with several types of contents in the lumen^36^; and ribosomes (Fig. 2a–c). We labeled these components *via* automated segmentation followed by manual refinement (Fig. 2d–f and Supplementary Video 2–4). Specifically, we applied the cylinder cross-correlation to segment actin filaments and MTs^37^, tomosegmemtv to segment membranous organelles such as ISGs and lysosomes^38^, and template matching to map ribosomes^39^. We define the segmentation unit as 60 nm for actin filaments and 100 nm for MTs.

We observed changes in the amounts of actin filaments and MTs in the tomograms of the cell periphery under different conditions (Fig. 2a–f), in accordance with previous fluorescence studies^25,40^. Specifically, both the volume ratio of actin filaments to the tomogram and the volume ratio of MTs to the tomogram significantly decrease in the first phase, indicating a nearly complete depolymerization. The volume ratio of actin filaments at the cell periphery after repolymerization does not change significantly compared to the basal conditions, in contrast to observations from randomly selected subsections of the cell in fluorescence images acquired above (Fig. 2g, h and Fig. 1h). This analysis provides a direct *in situ* quantification of remodeling of actin filaments and MTs at the cell periphery.

Actin filaments maintain cell structure by forming bundles parallel to the PM at the cell periphery^41^. They also regulate the transport and release of ISGs through a remodeling process, as proposed in previous studies using fluorescence microscopy^8,13^. We computed changes in the orientation of individual actin filaments relative to the PM (in the XY plane of a tomogram). Under basal conditions, 97% of the actin filaments are oriented to the PM at the angles of 0–45° (*ie*, parallel orientation). In the second phase, the amount of actin filaments oriented to the PM at the angles of 45–90° (*ie*, quasi-orthogonal orientation) increases from 3% to 15% (Fig. 2i); the amount of actin filaments with angles of 60–90° to the PM increases from 1% to 11%. This quantification of the reorientation after remodeling allows us to analyze various features of actin filaments as a function of their orientations to the PM under different conditions (below).

**Figure 3:**
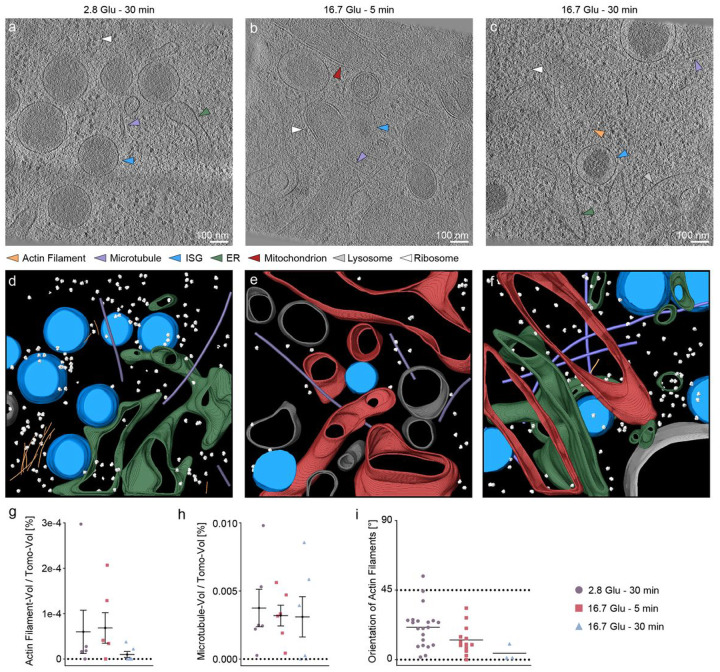
Quantitative analysis of actin filament and MT orientations in the cell interior during GSIS. **a, b, c** Reconstructed tomograms under basal conditions (2.8 Glu - 30 min; **a**), in the first phase (16.7 Glu - 5 min; **b**), and in the second phase (16.7 Glu - 30 min, **c**). Arrows indicate actin filaments (orange), MTs (violet), ISGs (blue), ER (green), mitochondria (red), lysosomes (silver), and ribosomes (white). **d, e, f** Respective segmentations with the same color code. Non-ISG membranes are shown in gray. **g**, **h** Fraction of actin filaments (**g**) or MTs (**h**) in each tomogram under different stimulation conditions. **h** Fraction of MTs in each tomogram under different stimulation conditions. **i** Orientation of actin filaments under different stimulation conditions. * indicates p<0.05. ** indicates p<0.01. *** indicates p<0.001. **** indicates p<0.0001 by one-way ANOVA. n = 18 tomograms, corresponding to six tomograms for each condition from three biologically independent experiments.

In tomograms of the cell interior, we distinguished different types of subcellular components, including actin filaments, MTs, endoplasmic reticulum (ER) as a continuous monolayer membrane^42,43^, mitochondria as a double layer membrane structure with a flat outer membrane and a curved interior membrane^35,44^, nuclear membrane^45^ and ribosomes^46^ (Fig. 3a–c). We labeled these components using the same methods as for the tomograms of the cell periphery (Fig. 3d–f and Supplementary Video 5–7). In the cell interior, we observed no substantial difference in either the amounts of actin filaments and MTs or the orientations of actin filaments relative to the PM under different conditions (Fig. 3g–i). Thus, we confirmed that the remodeling process does not appear to occur in the cell interior. The number of actin filaments is insufficient to demonstrate statistically significant differences between the different conditions (data not shown). Similarly, there are no statistically significant differences between distances spanned by the subcellular components studied here (data not shown).

In summary, peripheral actin filaments undergo a nearly complete depolymerization in the first phase and then a repolymerization during GSIS. Most importantly, more actin filaments are oriented quasi-orthogonally to the PM after remodeling than before remodeling (3% and 15% for the basal conditions and the second phases, respectively). In contrast, actin filaments in the cell interior do not undergo a remodeling process. Therefore, we proceed with the analysis of the peripheral tomograms only.

### The architecture of the actin filament network before and after remodeling

**Figure 4:**
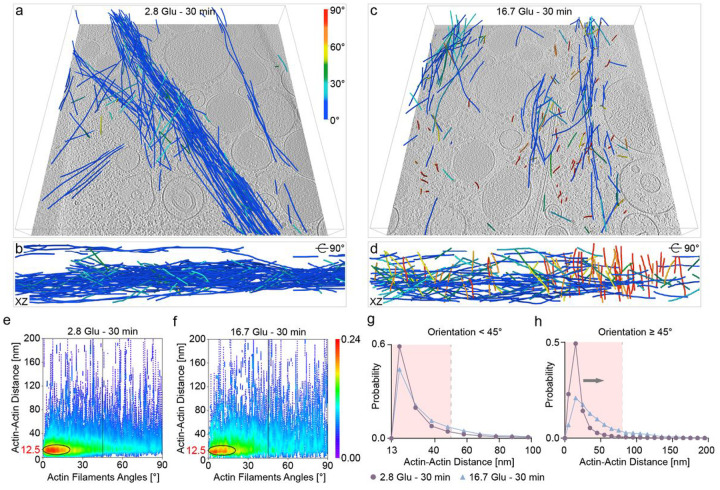
Quantitative analysis of actin filament organization at the cell periphery. **a-d** Actin filaments represented as a function of their orientation relative to the PM colored in a blue-to-red color map (shown in **a**) as a function of their orientation relative to the PM, before (**a, b**) and after (**c, d**) remodeling in two different views. **e, f** Density map of the distance between actin filaments as a function of the angle between actin filaments before (**e**) and after (**f**) remodeling. **g** Histogram of the distances between actin filaments whose orientation relative to the PM is less than 45° before and after remodeling. Bin size = 10. **h** Histogram of the distances between actin filaments whose orientation relative to the PM is greater than or equal to 45° before and after remodeling. Bin size = 10. Each point on the density map reflects the corresponding density values by calculating kernel density.

Tomograms of the cell periphery show a change in the architecture of the actin filament network during remodeling, despite a similar volume of actin filaments (Fig. 4a–d and Fig. 2g). We computed the Actin-Actin distance as a function of the actin filaments angles (Methods). Most actin filaments form bundles, presumably to fulfill their primary function of maintaining cell structure, with an angle of 0–15° and a distance of 12–13 nm, as found in filopodia and stress fibers in epithelial cells^32^ (Fig. 4e, f). However, we observe fewer bundles in the second phase compared to basal conditions (yellow-to-red circled region in Fig. 4e, f). In addition, actin filaments oriented parallel to the PM maintain the same distance distribution after remodeling, whereas actin filaments oriented quasi-orthogonally to the PM show a shift to longer distances in the second phase compared to the basal conditions (Fig. 4g, h). These results indicate that actin filaments mostly retain their bundled formation but partially reconfigure into a less compact arrangement that may be required to transport subcellular components from the cell periphery to the PM.

**Figure 5:**
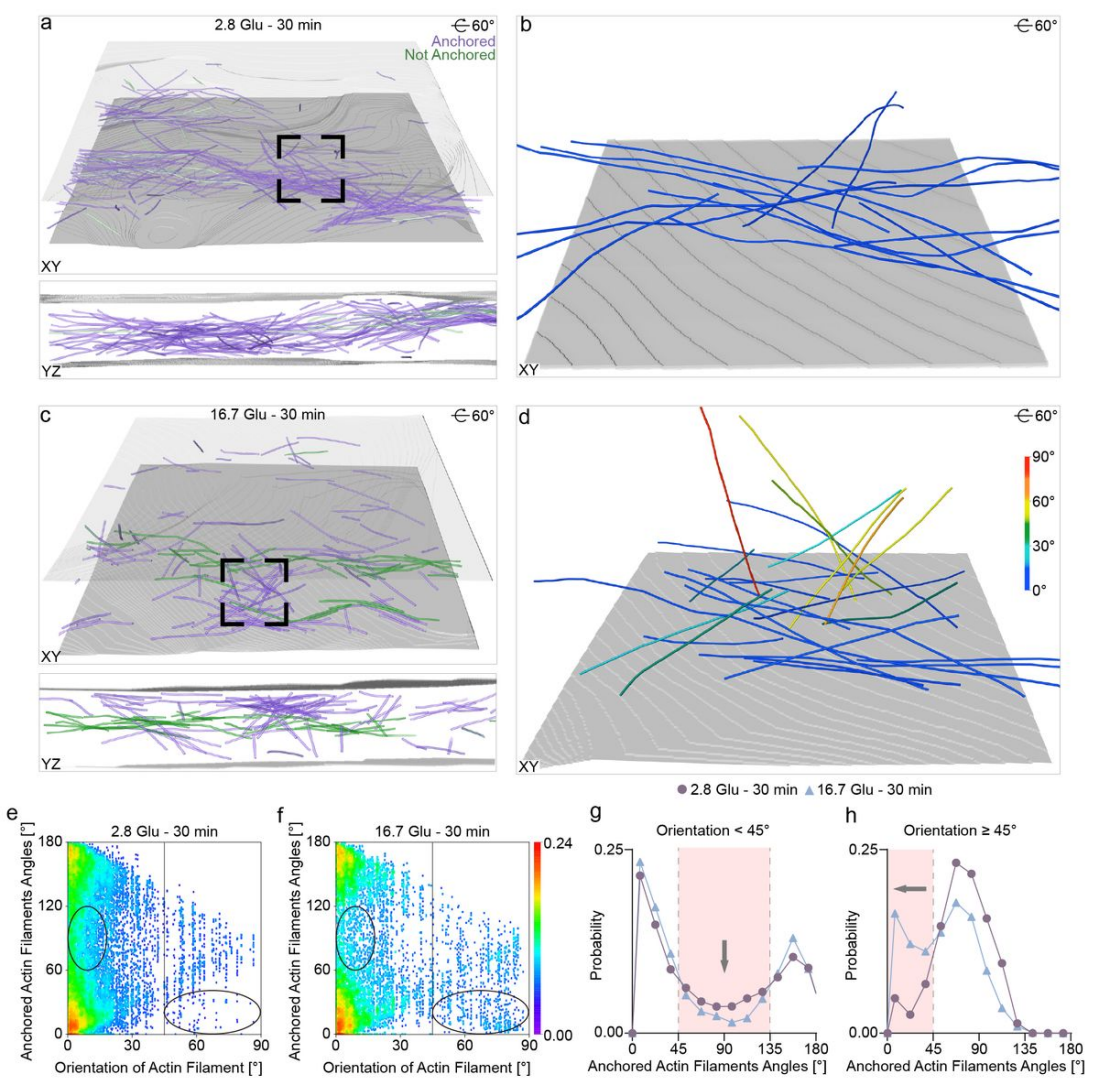
Quantitative analysis of PM-anchored actin filaments at the cell periphery. **a, c** 3D visualization of actin filaments (anchored: violet, not anchored: green) before (**a**) and after (**c**) remodeling. **b, d** Zoomed-in views of the actin filaments highlighted in a and c, represented as a function of their orientation relative to the PM in a blue-to-red color map, before (**b**) and after (**d**) remodeling. **e, f** Density map of the distance between PM-anchored actin filaments as a function of their orientation relative to the PM before (**e**) and after (**f**) remodeling. **g** Histogram of the angle between PM-anchored actin filaments whose orientation is less than 45° relative to the PM before and after remodeling. Bin size = 15. **h** Histogram of the angle between PM-anchored actin filaments whose orientation relative to the PM is greater than or equal to 45° before and after remodeling. Bin size = 15. Each point on the density map reflects the corresponding density values by calculating kernel density.

We manually labeled the PM and considered actin filaments within 60 nm of the PM as being anchored to the PM (Fig. 5a–d). The distance is defined as the shortest distance between the end-points of each actin filament and the PM; this distance threshold takes into account the length of the actin filament segmentation unit (60 nm). Under basal conditions and in the second phase, the height of the cell periphery (190.5 ± 16.9 nm and 258.4 ± 31.3 nm) remained constant, as well as the number and percentage of anchored actin filaments (263 ± 55 and 183 ± 68; 90% ± 4% and 71% ± 8%), as determined by a t-test (Extended Data Fig. 6). We calculated the angles between neighboring actin filament vectors that were anchored to the PM and mapped these angles against the orientations of the anchored actin filaments relative to the PM (Fig. 5e–h, Methods). The filament vector is defined as extending from the end-point near the PM to the end-point farther from it; neighboring filament vectors are defined as those anchored to the same PM whose end-points near the PM are within twice the length of the actin filament segmentation unit. Anchored actin filaments are primarily oriented parallel to the PM at the angles of 0–15° with the highest and next highest peaks of the filament alignment at the angles of 0–30° and 150–180° (Fig. 5e–f). This indicates a parallel alignment of the anchored actin filaments both with the PM and with each other (*ie*, bundles). Under basal conditions, actin filaments oriented quasi-orthogonally to the PM have the highest peak at 60–90°, suggesting a “netlike” architecture, as expected to act as a barrier that blocks ISGs from approaching the PM (Fig. 5g). In the second phase, actin filaments oriented parallel to the PM show a lower probability at angles of 60–90° compared to basal conditions (Fig. 5f, h). Actin filaments oriented quasi-orthogonally to the PM show a clear shift to smaller angles in the second phase compared with basal conditions (Fig. 5f, h). These findings suggest that actin filaments reorient quasi-orthogonally to the PM and parallel to each other after remodeling compared to basal conditions. This reorganization changes the actin filament network from a “netlike” to a “blooming” architecture, where radial projections emanate from anchor points at the PM (Fig. 5b, d). The “blooming” architecture is thought to act as transportation tracks to the PM, which is expected to facilitate the transport and release of ISGs at the PM. These results offer the first structural evidence for the previous hypothesis of actin filaments blocking and facilitating ISG release at the PM before and after remodeling, respectively^5,8,10^.

### Interaction of ISGs, actin filaments, and MTs at the cell periphery

**Figure 6:**
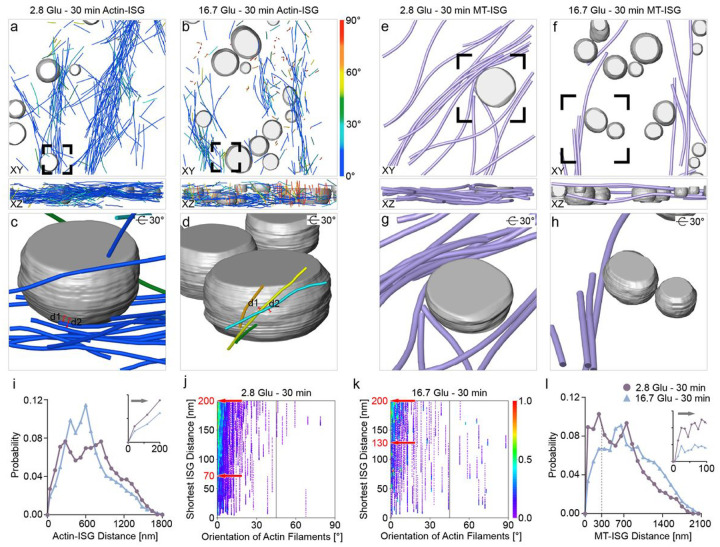
Quantification of the interaction between actin filaments and MTs with ISGs. **a, b** ISGs (gray) and actin filaments (same color code as in Fig. 4) visualized under basal conditions (**a**) and in the second phase (**b**). Results are shown in the XY (**a**) and XZ (**b**) planes. **c, d** Zoomed-in views of actin filaments in the vicinity of ISGs (gray) in positions highlighted by a black box in **a** and **b**, respectively. **c** Example distances d1 = 9 nm, d2 = 17.7 nm. **d** Example distances d1 = 15.4 nm, d2 = 9.1 nm. **e, f** ISGs (gray) and MTs (violet) are visualized under basal conditions (**e**) and in the second phase (**f**). **g, h** Zoomed-in views of the MTs (violet) with ISGs (gray) in positions highlighted by a black box under basal conditions (**g**) and in the second phase (**h**). **i** Plot of the distance distribution between actin filaments and ISGs under basal conditions and in the second phase. Bin size = 75. **j, k** Density maps of the shortest distance between actin filaments and ISGs as a function of their orientation relative to the PM under basal conditions (**j**) and in the second phase (**k**). **l** Plot of the distance distribution between MTs and ISGs under basal conditions and in the second phase. Bin size = 100. Each point on the density map reflects the corresponding density values by calculating kernel density.

Previous studies demonstrated that actin filaments interact with MTs to facilitate insulin secretion^10^. Specifically, the transport of ISGs occurs along MTs from the cell interior to the cell periphery, then along actin filaments from the cell periphery to the PM^10^. In tomograms of the cell periphery, we observed a few ISGs in vicinity of both actin filaments (Fig. 6a–d) and MTs (Fig. 6e–h). In addition, we observed several ISGs particularly close to the reoriented actin filaments in the second phase, suggesting a potential association with the transport process (Fig. 6b, d). These observations led us to quantitatively analyze the shortest distances between ISGs and actin filaments as well as between ISGs and MTs, as follows.

To characterize interactions between ISGs and actin filaments, we focused on variations in their shortest distances within 200 nm during GSIS. The shortest distance is calculated as the distance between the resampled points of actin filaments (at 4 nm intervals) and the nearest ISG surface (Methods). This distance threshold is estimated by considering the length of the actin filament segmentation unit (60 nm) and the lengths of myosin V and other unknown associated proteins (approximately 55 nm for the structurally defined part of myosin V^16^ and an additional tolerance of 85 nm to account for the uncertainty resulting from the unknown structure of 268 residues of myosin V and unknown associated proteins). The probability for the shortest distance to be within 200 nm decreases in the second phase compared with basal conditions (Fig. 6i). We mapped the shortest distances along different orientations of actin filaments relative to the PM. Actin filaments oriented parallel to the PM at orientations less than 15° have a larger shortest distance to ISGs in the second phase (130–200 nm) compared with basal conditions (70–200 nm) (cyan in Fig. 6j, k). These results indicate a further spatial arrangement of actin filaments relative to ISGs after remodeling compared with basal conditions, which is expected for the subsequent transport and release of ISGs.

The critical role of MT-dependent transport of newly generated ISGs under glucose stimulation has been well established^10,47^. MTs have also been proposed to negatively regulate insulin secretion at the cell periphery under basal conditions, based on a fluorescence microscopy observation of restricted ISG movements during MT stabilization by drugs^17^. Here, we calculated the MT-ISG distance as the distance between the resampled MTs (at 4 nm intervals) and the nearest ISG surface (Methods). We observe the highest peak region starting at 20 nm and centered at 200 nm under basal conditions (Fig. 6l), with a significant shift to longer distances in the second phase. This observation provides a structural perspective at the nanoscale on the previously proposed negative regulation of MTs for ISG release under basal conditions^17^. Specifically, MTs “trap” instead of “transport” ISGs, because the transportation requires Kinesin-1, which has a size of approx. 60 nm. Moreover, this effect is significantly reduced in the second phase.

**Figure 7:**
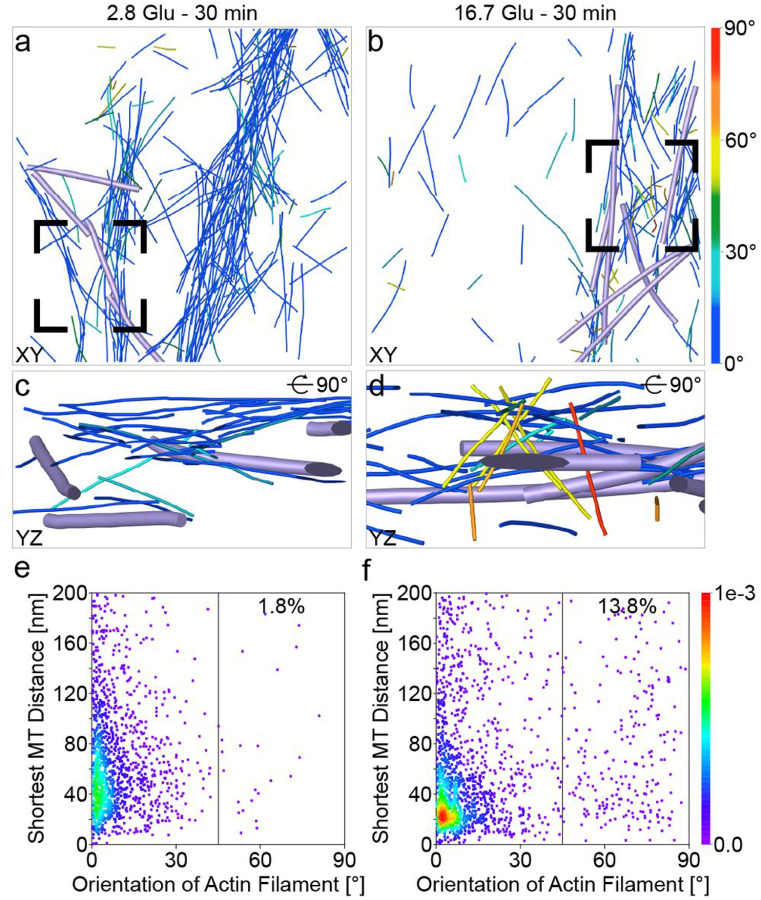
Distance between actin filaments and MTs during GSIS. **a, b** Actin filament (same color code as in Fig. 4) and MT (violet) organization shown in the XZ plane under basal conditions (**a**) and in the second phase (**b**). **c, d** Zoomed-in views of actin filaments in the vicinity of ISGs (gray) in positions highlighted by a black box in **a** and **b**, respectively. **e, f** Density maps of the shortest distance between actin filaments and MTs under basal conditions (**e**) and in the second phase (**f**). Each point on the density map reflects the corresponding density values by calculating kernel density.

Lastly, for each actin filament, we computed the distance to the closest MT (shortest MT distance, Methods) to investigate their interactions (Fig. 7). The total volume of actin filaments and MTs remains constant during GSIS. Actin filaments oriented parallel to the PM at the angles of 0–15° tend to be closer to MTs, as indicated by their shortest MT distances in a narrower range in the second phase (20–300 nm) compared to basal conditions (20–800 nm; red to green in Fig.7e, f). Actin filaments oriented quasi-orthogonally to the PM also tend to be located closer to MTs during the second phase compared to basal conditions. In summary, actin filaments parallel and quasi-orthogonal to the PM are both closer to MTs during the second phase than under basal conditions.

## Discussion

Actin filaments are known to regulate ISG transport and release under glucose stimulation. Because of the limited resolution of fluorescence microscopy used in previous studies, actin remodeling has been characterized primarily as a “strong distribution” under basal conditions, a “diminished amount” during the first phase, and a “recovery” during the second phase^48^. Here, we applied multimodal imaging to map INS-1E β-cells under basal conditions, as well as in the first and second phases of insulin secretion. At the whole-cell level, we captured the biphasic insulin secretion and actin meshwork organization using TIRF (Extended Data Fig. 1 and Video 1), measured the G-/F-actin ratio by western blot (Extended Data Fig. 2) and revealed changes in the length and number of junctions in the actin meshwork in randomly selected subsections of the β-cells by SIM (Fig. 1). At the subcellular level, we mapped actin remodeling at the periphery and interior of β-cells by cryo-ET (Fig. 2 and Fig. 3). Our work provides the first *in situ* structure of actin remodeling at the nanoscale, as well as a quantitative analysis of changes in the architecture, alignment and interaction of the actin filaments during GSIS. We demonstrate that actin remodeling occurs at the cell periphery but not in the cell interior. We establish the model for actin remodeling at the cell periphery as follows.

**Figure 8:**
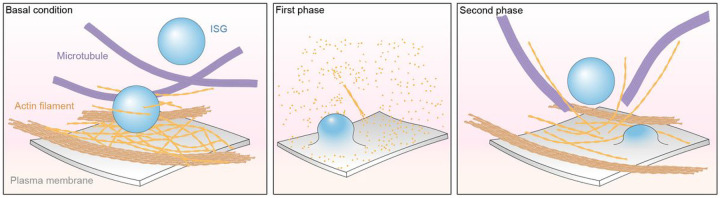
Schematic representation of the model for actin remodeling at the cell periphery during GSIS. The actin filaments, MTs, and ISGs at the cell periphery during GSIS under basal conditions, first phase, and second phase are depicted.

Under basal conditions (Fig. 8, left panel), actin filaments are mostly oriented parallel to the PM at the angles of 0–45°, forming bundles that maintain the cell structure (Fig. 4). Actin filaments anchored to the PM form a “netlike” architecture that presumably prevents ISGs from approaching the PM (Fig. 5). MTs adopt a close conformation surrounding ISGs, potentially limiting their movement (Fig. 6). Thus, we present a structural perspective on how actin filaments and MTs act as barriers to block the transport and release of ISGs under basal conditions^13,17,49^. In the first phase (Fig. 8, middle panel), actin filaments are nearly completely depolymerized, MTs and ISGs are almost absent, indicating rapid insulin secretion. In the second phase (Fig. 8, right panel), most actin filaments remain organized in bundles, presumably to fulfill their primary function of maintaining cell structure. Actin filaments reconfigure into a new network after repolymerization, leading to three major structural rearrangements that potentially facilitate the transport and release of ISGs from the PM: (i) approximately 12% of actin filaments reorient themselves quasi-orthogonally to the PM at the angles of 45–90° (Fig. 2); (ii) the actin filament network mostly remains as cell-stabilizing bundles but partially reconfigures its architecture into a less compact arrangement (Fig. 4); (iii) actin filaments anchored at the PM reorganize from a “netlike” to a “blooming” architecture, which is apparently required for transporting ISGs from the cell periphery to the PM (Fig. 5).

The rearrangements of the actin filament network serve the functional role of regulating insulin secretion. They precede the transport of ISGs by their reorientations quasi-orthogonal to the PM and less compact packing as well as the release of ISGs by the formation of a “blooming” architecture of actin filaments anchored to the PM. Additionally, our analysis reveals changes in interactions among actin filaments, MTs, and ISGs during GSIS. Previous fluorescence studies have led to a proposal that both actin filaments and MTs regulate ISG transport and release by demonstrating limited ISG movements under chemical stabilization of either actin filaments or MTs^13,17^. Here, we show that actin filaments and MTs are further away from ISGs in the second phase compared to the basal conditions (Fig. 6), while being closer to each other (Fig. 7), as expected for the subsequent transport and release of ISGs.

We now discuss the limitations of our work. First, although INS-1E β-cells can capture biphasic insulin secretion and actin remodeling under glucose stimulation (Extended Data Fig. 1, Extended Data Fig. 2 and Supplementary Video 1), their signaling and metabolism pathways differ from those in primary β-cells^21^. Thus, it would be valuable to map primary β-cells using cryo-ET in healthy and diabetic states to depict variations in their structural and functional mechanisms. Second, we only mapped the periphery and interior of the β-cell due to the limitations of cryo-ET in mapping relatively thick cells. Future work shall cover more subcellular neighborhoods to obtain a more complete picture of actin remodeling throughout the β-cell. For instance, Ca^2+^ microdomain beneath the PM may be important in the normal regulation of insulin secretion in β-cells^50^. Another future focus should be on the secretion sites near the PM, to gain deeper insights into the secretion mechanism. Indeed, mapping the interactions between actin filaments and other organelles throughout the entire β-cell would provide valuable insights into their importance for ISG positioning, transport and release^51^. Third, we analyzed the structure of actin filaments anchored to the PM using a distance threshold. It is known that actin filaments are anchored to the PM *via* physical connections with focal adhesion complexes at the PM^8^. As an example, gold particle labeling can be used to detect these complexes in order to reveal interactions between focal adhesions and actin filaments during remodeling, as well as to better understand the molecular mechanisms involved in insulin secretion.

In summary, our study presents the structure and quantitative analysis of actin remodeling at the nanoscale in both the periphery and interior of β-cells, including changes in the architecture of the actin filament network, the alignments of actin filaments and the interaction among actin filaments, MTs, and ISGs during GSIS. These analyses contribute to our understanding of actin remodeling and its role in the regulation of biphasic insulin secretion in pancreatic β-cells.

## Materials And Methods

### 1. INS-1E β-cells culture, treatment, and plasmid transfection

Clonal rat INS-1E β-cells (gifted from P. Maechler’s laboratory at the University of Geneva^52^) were cultured in RPMI-1640 GlutaMAX^™^−1 medium (Thermo Fisher Scientific) containing 11.1 mM glucose, supplemented with 10 mM HEPES (Thermo Fisher Scientific), 5% heat-inactivated fetal bovine serum (FBS) (Thermo Fisher Scientific), 100 U/ml penicillin and streptomycin (Thermo Fisher Scientific), 1 mM sodium pyruvate (Sigma-Aldrich) and 50 μM β-mercaptoethanol (Sigma-Aldrich). Cells detached by 0.25%trypsin (Thermo Fisher Scientific) were seeded at a density of 4 × 10^4^ cells/cm^2^ on either Falcon culture dishes (Corning) or Poly-L-ornithine (Sigma-Aldrich) coated glass-bottom dishes (ibidi) and 6-well plates (Corning), and were grown at 37°C in 5% CO_2_. The culture medium was changed four days after cell seeding, and cells were passaged every seven days to maintain the cell line.

To achieve different cellular phenotypes under different conditions, INS-1E β-cells were exposed for 30 min in KREBS solution containing 2.8 mM glucose as the basal conditions, then changed into the KREBS solution containing 16.7 mM glucose and kept for 5 min to simulate the first phase of GSIS, and kept for 30 min to simulate the second phase of GSIS.

For cell transfection, INS-1E β-cells were used 48 hours post-cell seeding, and transfected with plasmids of pEGFP-C1.LifeAct-EGFP and pEGFP-N1.NPY-mCherry (modified from RRID: Addgene_58470 and RRID: Addgene_67156, respectively) using lipofectamine 3000 reagent (2 μl lipofectamine and P3000 per 1mg of plasmid, Thermo Fisher Scientific) to label the ISGs and actin filaments. The transfected INS-1E β-cells were then cultured for two days till use.

### 2. Culture and vitrification of INS-1E β-cells on EM grid

Quantifoil R 1/4 Au grids with 200 mesh holey carbon film (Quantifoil Micro Tools GmbH) were firstly coated with 0.5% Poly-L-ornithine (Sigma-Aldrich) diluted by PBS (Thermo Fisher Scientific) for 30 min. 4–6 coated grids were placed with carbon film upwards in 35-mm Falcon dishes containing 1 mL of complete RPMI 1640 medium, before adding the INS-1E β-cell suspension to reach the density of 4 × 10^4^ cells/cm^2^. The cells were grown on the grids for 48 hours, and then the grids were blotted from the backside for 10s using a Vitrobot Mark IV (Thermo Fisher Scientific) with 10% humidity, and plunge-frozen in ethane liquid. Subsequently, the vitrified samples were transferred into cryo-EM boxes and stored in liquid nitrogen before FIB-milling or cryo-ET data collection.

### 3. Live-cell fluorescence imaging

To record the INS-1E live-cell images during the time of GSIS, cells were grown on 35 mm glass-bottom dishes (ibid), transfected with plasmids to label ISGs and actin filaments, and stimulated with glucose as described above. The movies were acquired using wide-field TIRF mode under light microscope system at 37°C in 5% CO_2_. This light Microscopy was performed on a Nikon Ti2-E with TIRF equipped with a Prime 95B Scientific CMOS using an Apo TIRF 60× Oil 1.49 objective. Fluorescence channels were Hoechst 33342 (Ex 345–355 nm, Em 450–460 nm), EGFP (Ex 483–493 nm, Em 502–512 nm) and mCherry (Ex 582–592 nm, Em 605–615 nm). Finally, the resolution of TIRF data is x-122.2 nm, y-122.2 nm.

### 4. Western blot

The ratio of G- and F-actin was performed following the manufacturer’s protocols (Cytoskeleton). Protein samples were separated by SDS-PAGE and transferred to PVDF membrane (Thermo Fisher) with known amounts of actin used for quantitation in G-/F-actin ratio experiments. Membranes were with 0.1% Tween 20 (Merck). Incubate with the primary antibody (rabbit) provided in the kit overnight at 4 °C. Then incubate with the secondary antibody (Abcam, anti-rabbit, 1:5000). Finally, the membrane was exposed after incubation in a dark environment with ECL substrate for 1 min (Bio-rad).

### 5. Super-resolution fluorescence microscopy of fixed INS-1E β-cells

To prepare the fixed INS-1E β-cell samples, cells were grown on a glass coverslip (ibidi), transfected with plasmids to label ISGs and actin filaments, and stimulated with glucose to acquire three different conditions (basal conditions, the first phase and second phase of GSIS) as described above. Then the samples were washed with 1xPBS three times at room temperature, and incubated with 4% paraformaldehyde for 20 min. Sequentially the cells were immediately washed three times with 1×PBS and incubated in 2 mL of Hoechst 33342 working buffer (1 μg/mL, Cell Signaling) for 5 min. After that, samples were washed three times with 1xPBS, and the glass coverslip was applied with one drop of ProLong^™^ Glass Antifade Mountant (Thermo Fisher Scientific) and transferred onto the glass slide. Finally, the samples were kept at room temperature for 48 hours and later stored at −20°C in the dark. The super-resolution fluorescence images were collected in SIM (structured illumination microscopy) mode on Zeiss Elyra 7 with Lattice SIM, equipped with a PCO edge 4.2 sCMOS camera using a Plan-Apochromat 63x/1.4 Oil DIC M27. Fluorescence channels were Hoechst 33342 (Ex 345–355 nm, Em 450–460 nm), EGFP (Ex 483–493 nm, Em 502–512 nm) and mCherry (Ex 582–592 nm, Em 605–615 nm). Finally, the resolution of the SIM data is x-31.3 nm, y-31.3 nm, and z-90.9 nm.

### 6. Fluorescence image analysis

To quantify the total length and network junction of actin meshwork, a total of 72 subsections with each containing an area of 0.97 μm^2^ were produced from the super-resolution fluorescence images of INS-1E β-cells in different conditions (including 24 subsections of basal conditions, first phase and second phase of GSIS, respectively), by using the cut tool in the Adobe Photoshop CC 19.1.19. All these subsections were subjected to a cascade of processing using Image J (v1.53f51), firstly the actin is filtered using the “Threshold-default” tool, then skeletonized using the “skeletonize” tool, ultimately, the information of actins was determined using “Analyze skeleton” tool, including the total length and the network junction. Based on “Analyze skeleton”, we here only considered meaningful actins whose length is larger than one pixel for the following measurement^26^. Analysis was repeated for six independent cell images under each condition.

### 7. Cryo-FIB milling

Cryo-FIB milling was carried out following similar procedures as previously described^53^, using dualbeam FIB-SEM microscopes Aquilos 2 Cryo FIB (Thermo Fisher Scientific). Grids holding frozen cells were clipped into autogrid support rings with a cutout region to facilitate shallow-angle cryo-FIB milling. The Autogrids were mounted onto the cryo-FIB AutoGrid shuttle and transferred to the cryo-stage at liquid nitrogen temperatures. Before the milling procedure, grids were sputter-coated with platinum for the 30s (10 mA) and subsequently sputter-coated with organometallic platinum using the gas injection system (GIS, Thermo Fisher Scientific) for 8 s to improve conductivity and remove artifacts. Samples were milled by gallium ion beam at 30 kV with a stage tilting angle of 17–19° to generate 10–12 μm wide lamellas, the initial rough milling was done under 0.5 nA high currents, then the current was gradually decreased in stepwise manner to 30 pA for fine milling and final polishing. Electron beam at 2 kV/13 pA or 5 kV/25 pA was used for SEM imaging during the milling process. In total, 18 lamellas from randomly chosen cells were used for the following cryo-ET data collection.

### 8. Cryo-ET acquisition and reconstruction

The grids holding either frozen INS-1E β-cell or lamella samples produced by FIB-milling were loaded in a Titan Krios G3 or G4 TEM (Thermo Fisher Scientific). The Titan G3 TEM was equipped with a 300-kV field-emission gun, a post-column energy filter (Gatan), and a 5760 × 4092 K3 Summit direct electron detector (Gatan), operated using SerialEM^54^. Low-magnification images were captured at 3600×. High-magnification tilt series were recorded in counting mode at 26000x (calibrated pixel size of 0.3353 nm). The Titan Krios G4 TEM (Thermo Fisher Scientific) was equipped with a 300-kV C-FEG field-emission gun, a post-column energy filter (Selectris), and a 4096 × 4096 direct electron detector (Falcon4), operated using Tomography (Thermo Fisher Scientific). Low-magnification images were captured at 5600×. High-magnification tilt series were recorded in counting mode at 42000x (calibrated pixel size of 0.3028 nm). For cell periphery samples, tilt series were collected with ± 60° tilt range, 2° step. 24 tilt-series, collected from 16 different cells were collected in total. For lamella samples, a similar scheme covering 120° with 2° increments starting from ~ 10° compensating the pre-tilt of lamella was applied. 18 tilt-series, collected from 18 different cells were collected in total. All 42 tomograms were recorded at a total dose of 110–140 e-/Å2 using a dose-symmetric tilt scheme^55^ and a target defocus range of −3 to −7 μm (Supplementary Tables 1–2).

Data preprocessing including motion correction by MotionCor2^56^ and the dose-filtering step^57^ was performed using the TOMOMAN package which could execute on MATLAB (2019b) software (https://github.com/williamnwan/TOMOMAN). Tilt-series were aligned by patch tracking in IMOD^58^ and reconstructed to 4x binned tomograms (with a pixel size of 13.4 Å and 12.1 Å for cell periphery and lamella, respectively) using weighted-back projection. Tomograms were then denoised by the cryoCARE algorithm (https://github.com/juglab/cryoCARE_T2T) for better segmentation and visualization.

### 9. Filament and membrane segmentation

The above mentioned 4× binned tomograms were used for the segmentation. Correlative volumes of membrane positions were detected and generated automatically using tomosegmemtv^38^ and imported to Amira software (Thermo Fisher Scientific) for manual refinement and segmentation. Actin filaments and MTs were traced automatically in Amira software using an automated segmentation algorithm, which adopted a cylinder as a template^37^ and implemented in the X-Tracing extension. The cylindrical templates were generated with a length of 60 nm or 100 nm and diameters of 8 nm or 15 nm for the actin filaments or MTs, respectively. After the automated segmentation algorithm, the segmented results were manually checked by the tomogram intensity map. The identification of the PM positions within the tomogram is achieved through a manual segmentation process, whereby both intracellular and extracellular regions are recognized to create a PM mask.

### 10. Data analysis

Data analysis was performed using in-house Python scripts using coordinates of actin filaments and MTs, PM mask volumes, and ISG mask volumes, which were exported from Amira software. First, we adjusted the coordinates based on the offset of each tomogram. Next, we resampled actin filaments and MTs with 4 nm intervals; this interval has been previously shown to provide a reasonable fit in other analyses^32^. The Actin-Actin distance represents the distance between the centerline of two actin filament segments, which was calculated as the Euclidean distance between each resampled point on the actin filament and its nearest resampled points on all other actin filaments (Fig. 4). The actin filaments angles represent the relative orientations between each two actin filaments. This angle was calculated as the angle between the vectors formed by two end-points of the actin filament and the vectors formed by two end-points of all other filaments (Fig. 4). The anchored actin filament angles represent the relative orientations between two nearby actin filaments anchored to the PM. For each anchored actin filament, the neighboring filaments are identified if: 1) they were anchored to the same PM, and 2) the distance between their end-points near the PM was less than 120 nm, which is twice the length of the actin filament segmentation unit (60 nm). The angle was then calculated between each anchored filament vector extending from the end-point near the PM to the end-point farther from it and all other nearby anchored filament vectors (Fig. 5). The Actin-ISG distance was calculated as the Euclidean distances of each resampled point of actin filaments to the nearest ISG surface (Fig. 6). The MT-ISG distance was calculated in the same way as the Actin-ISG distance (Fig. 6). The shortest MT distance of actin filament was calculated as the Euclidean distance between each resampled point on the actin filament and its nearest resampled points on all MTs (Fig. 7).

All statistical analysis was undertaken using GraphPad Prism (v9.4.1) and OriginPro (v9.8.0.200). Statistical significance tests for the total length and network junction of actin meshwork, the analysis of both cell periphery and interior of the cell ratio of actin filament-vol/tomo-vol and MT-vol/tomo-vol, and orientation of actin filaments at the cell interior were calculated using one-way ANOVA test, followed by Tukey’s multiple comparisons tests. Statistical significance tests for the orientation of peripheral actin filaments, the average cell height, the number of anchored actin filaments, and the percentage of anchored actin filaments by total actin filaments were calculated using an F-test and t-test. Values are reported as the mean values. The error bars represent SEMs unless otherwise stated.

## Data Availability

Data is available from the corresponding authors upon request.
